# Parent Mental Health and Family Coping over Two Years after the Birth of a Child with Acute Neonatal Seizures

**DOI:** 10.3390/children9010002

**Published:** 2021-12-22

**Authors:** Linda S. Franck, Renée A. Shellhaas, Monica E. Lemmon, Julie Sturza, Marty Barnes, Trisha Brogi, Elizabeth Hill, Katrina Moline, Janet S. Soul, Taeun Chang, Courtney J. Wusthoff, Catherine J. Chu, Shavonne L. Massey, Nicholas S. Abend, Cameron Thomas, Elizabeth E. Rogers, Charles E. McCulloch, Hannah C. Glass

**Affiliations:** 1 Department of Family Health Care Nursing, University of California San Francisco, San Francisco, CA 94143, USA; trishabarbour@gmail.com; 2 Department of Pediatrics, University of Michigan, Ann Arbor, MI 48109, USA; shellhaa@med.umich.edu (R.A.S.); jmigrin@umich.edu (J.S.); elizhill@med.umich.edu (E.H.); 3 Departments of Pediatrics and Population Health Sciences, Duke University School of Medicine, Durham, NC 27710, USA; monica.lemmon@duke.edu; 4 Casey’s Circle, Austin, TX 78717, USA; mstockton@gmail.com; 5 Hand to Hold, Austin, TX 78750, USA; katrina@handtohold.org; 6 Department of Neurology, Boston Children’s Hospital, Boston, MA 02115, USA; janet.soul@childrens.harvard.edu; 7 Harvard Medical School, Boston, MA 02115, USA; cjchu@mgh.harvard.edu; 8 Department of Neurology, Children’s National Hospital, Washington, DC 20010, USA; TChang@childrensnational.org; 9 Department of Neurology, George Washington University School of Medicine and Health Sciences, Washington, DC 20052, USA; 10 Departments of Neurology and Pediatrics, Stanford University, Palo Alto, CA 94304, USA; wusthoff@stanford.edu; 11 Department of Neurology, Massachusetts General Hospital, Boston, MA 02114, USA; 12 Departments of Pediatrics, Children’s Hospital of Philadelphia, Philadelphia, PA 19104, USA; MASSEYSL@chop.edu (S.L.M.); ABEND@chop.edu (N.S.A.); 13 Departments of Neurology and Pediatrics, Perelman School of Medicine, University of Pennsylvania, Philadelphia, PA 19104, USA; 14 Division of Neurology, Cincinnati Children’s Hospital Medical Center, Cincinnati, OH 45229, USA; Cameron.Thomas@cchmc.org; 15 Department of Pediatrics, University of Cincinnati College of Medicine, Cincinnati, OH 45267, USA; 16 Department of Pediatrics, UCSF Benioff Children’s Hospital, University of California San Francisco, San Francisco, CA 94143, USA; Elizabeth.Rogers@ucsf.edu; 17 Department of Epidemiology and Biostatistics, University of California San Francisco, San Francisco, CA 94143, USA; Charles.McCulloch@ucsf.edu; 18 Departments of Neurology, Pediatrics, Epidemiology and Biostatistics, Weill Institute for Neuroscience, UCSF Benioff Children’s Hospital, University of California San Francisco, San Francisco, CA 94143, USA; Hannah.Glass@ucsf.edu

**Keywords:** parental mental health, family impact, neonatal seizures, epilepsy, child development

## Abstract

Little is known about parent and family well-being after acute neonatal seizures. In thus study, we aimed to characterize parent mental health and family coping over the first two years after their child’s neonatal seizures. Parents of 303 children with acute neonatal seizures from nine pediatric hospitals completed surveys at discharge and 12-, 18- and 24-months corrected age. Outcomes included parental anxiety, depression, quality of life, impact on the family, post-traumatic stress and post-traumatic growth. We used linear mixed effect regression models and multivariate analysis to examine relationships among predictors and outcomes. At the two-year timepoint, parents reported clinically significant anxiety (31.5%), depression (11.7%) and post-traumatic stress (23.7%). Parents reported moderately high quality of life and positive personal change over time despite ongoing challenges to family coping. Families of children with longer neonatal hospitalization, functional impairment, post-neonatal epilepsy, receiving developmental support services and families of color reported poorer parental mental health and family coping. Parents of color were more likely to report symptoms of post-traumatic stress and positive personal change. Clinicians caring for children with neonatal seizures should be aware of lasting risks to parent mental health and family coping. Universal screening would enable timely referral for support services to mitigate further risk to family well-being and child development.

## 1. Introduction

Neonatal seizures are a medical emergency. The prognosis following acute neonatal seizures can be difficult to predict and may include epilepsy and cerebral palsy [1,2]. The birth of a child with a medical condition that carries a risk of death, long-term health problems, or an uncertain prognosis is an extremely stressful experience for parents [3]. Parents of infants requiring neonatal intensive care unit (NICU) hospitalization are at risk for persistent anxiety, depression, post-traumatic stress and poor quality of life, which can adversely impact the long-term health and development of the child and family [4,5].

We previously characterized the association between acute symptomatic neonatal seizures and parent and family well-being at discharge from neonatal hospitalization [6]. More than half of parents experienced clinically important symptoms of anxiety, and almost one-third experienced clinically important symptoms of depression. We found that higher maternal education, hypoxic-ischemic encephalopathy (HIE) seizure etiology and older infant age at discharge were associated with increased symptoms of anxiety and depression and worse family coping. However, parental mental health and family coping beyond the neonatal period has not been characterized for families of children with neonatal seizures.

Given the strong influence of parent and family well-being on child mental health and development [7,8], and the high risk for adverse neurodevelopment after neonatal seizures [2], it is imperative to examine parent and family well-being over time for infants with neonatal seizures. Therefore, the purpose of this study was to describe parent and family well-being over the first two years of life of a child with neonatal seizures. We also examined the association of parent and child characteristics with parent and family well-being over time. Greater understanding of the trajectory of family well-being can inform strategies to improve family well-being and promote infant development.

## 2. Materials and Methods

Longitudinal examination of the well-being of families was a pre-specified secondary aim of a comparative effectiveness study examining the safety of early discontinuation of antiseizure medication after acute symptomatic neonatal seizures [9]. Families were enrolled at one of nine United States (US) children’s hospitals. The study design, outcome measures and analyses were informed by a Parent Advisory Panel. The study was approved by the Institutional Review Board (IRB) of the University of Michigan (HUM00114541; 7 August 2016) and the IRBs at all other study sites. Parental written informed consent was obtained prior to enrollment.

### 2.1. Participants

Approximately half of the participants were enrolled during NICU admission, with the remainder recruited prior to age 24 months from the first Neonatal Seizure Registry cohort [10] or from outpatient clinics at a participating institution. Enrollment criteria were: (1) neonate with EEG-confirmed seizures or neonate treated with anti-seizure medication for diagnosed or suspected seizures, (2) the seizures had an acute symptomatic etiology (i.e., hypoxic-ischemic encephalopathy [HIE], ischemic stroke, intracranial hemorrhage [ICH] or other brain injury) and (3) the infant survived the neonatal seizure admission. Excluded were neonates with transient cause for seizures (e.g., hyponatremia, hypocalcemia, hypoglycemia without brain injury) or neonatal onset genetic epilepsy syndromes. Characteristics of these infants, along with analyses related to neonatal seizure treatment and developmental outcomes, have been reported [9].

One parent or other legal guardian per family completed a suite of validated survey instruments near the time of discharge from the NICU and when their child reached 12-, 18- and 24-months corrected age. Parents completed the surveys online or by telephone interview with a trained research assistant.

### 2.2. Measures

Measures of parent and family well-being included the following well-validated scales. Three scales measured parental mental health symptoms or family coping difficulties: the Hospital Anxiety and Depression Scale (HADS) [11]; Impact of Events (IES) post-traumatic stress symptom scale (revised) [12]; Impact On Family (IOF) scale (revised) [13]. Parental quality of life was measured using the World Health Organization Quality of Life (WHOQOL-BREF) scale [14] and positive personal change after experiencing significant stress or trauma was measured with the Post-traumatic Growth Inventory (PTGI) [15].

Demographic variables included child insurance type (public or private), child race and ethnicity and parent-reported maternal education level. These were obtained by medical record review or parent report. Clinical course variables obtained by medical record review included gestational age at birth (with preterm birth defined as <37 weeks completed gestation), complex medical condition (defined as preterm, surgical congenital heart disease, or required extracorporeal membrane oxygenation), seizure etiology (i.e., HIE, ischemic stroke, ICH or other acute brain injury), discharge on antiseizure medication and age at discharge from the neonatal seizure admission.

Variables related to the child’s neurological outcome included diagnosis of epilepsy or cerebral palsy, and functional neurodevelopment at corrected age 24 months as measured with the Warner Initial Developmental Evaluation of Adaptive and Functional Skills (WIDEA-FS) [16]. A child was considered to have functional impairment when their WIDEA-FS total score was more than two standard deviations below the mean for age. Post-neonatal epilepsy was defined per International League Against Epilepsy criteria [17] and determined by parent report, corroborated by local study investigator systematic chart review. Parents also reported whether their child received developmental support services (e.g., physical therapy, occupational therapy or speech therapy).

### 2.3. Statistical Analysis

Study personnel who coded clinical variables were blinded to the results of parent well-being assessments. All analyses were conducted with SAS^TM^ Software version 9.4 (Cary, NC, USA). Univariate statistics described the sample. To examine changes in well-being over time, we used a series of linear mixed effect regression models, one for each well-being outcome, utilizing restricted maximum likelihood fitting and the Kenward–Roger degree of freedom adjustments. We included person-specific intercepts and slopes over time, and controlled for institution, time point of measurement and which caregiver (i.e., mother, father or another legal guardian) responded. Factors of interest were maternal education and child race and ethnicity (Black, Indigenous or People of Color [BIPOC] vs. white or non-Hispanic), insurance type (public vs. private), neonatal seizure etiology, length of neonatal seizure hospital stay, antiseizure medications maintained (vs. discontinued) upon hospital discharge, functional impairment, receiving developmental support services, diagnosed with epilepsy by the measurement timepoint and diagnosed with cerebral palsy by 24 months. Backward stepwise selection was used to determine which factors of interest were retained in the final models.

We also conducted a single, multivariate analysis utilizing all six outcomes (HADS anxiety, HADS depression, IES, IOF, WHOQOL-BREF and PTGI) measured at discharge and 12, 18 and 24 months. To make the outcomes more comparable in this multivariate analysis, we first converted them to Z-scores and aligned the directionality so that lower scores indicated better well-being for all outcomes. The multivariate analysis allowed us to assess whether an exposure had similar or differential effects on the well-being outcomes. When the effect was similar, it allowed us to pool across outcomes to increase precision.

Linear mixed models were also used for these analyses. However, in this case, we used empirical (robust) standard errors to accommodate arbitrary forms of correlations across the six well-being outcomes and across the three time points. We included predictors of outcome type (to allow for different mean values across outcomes), exposure (patient/parent demographics, neonatal seizure characteristics, clinical course parameters) and data collection time point, as well as the interaction of exposure and outcome type (to assess similarity of the association across the six outcomes). We first ran the full model, then removed non-significant terms, one by one, in an order that took statistical significance and practical/clinical importance into account.

Once non-significant terms were removed, we considered each of the exposures in turn. If an interaction with parent or family well-being outcome was present (indicating that differences between categories of the exposure were not the same across outcome types), we then reported interaction *p*-values and presented the results graphically by showing the effect estimates plotted versus outcome type (“interaction plots”). If there was not an interaction, then we calculated overall estimates, essentially averaging over outcome types.

Given the large number of comparisons, we report results with *p* < 0.01 as statistically significant and results with 0.01 < *p* < 0.05 as nominally significant.

## 3. Results

### 3.1. Sample Characteristics

Of the 305 infants enrolled (n = 150 enrolled during neonatal admission, n = 155 enrolled at later clinic visit), two were excluded because they did not meet entry criteria. The final sample included 303 primary caregivers. In 227 (75%) cases, the respondent at all time points was the mother and, in 27 (9%) cases, it was the father. In the remaining 49 cases, the respondent varied across the timepoints between mother and father or, in a few cases, another legal guardian (n = 5 infants; six time points). Table 1 provides a full sample description. Most infants were term or near-term at birth and were approximately two weeks of age at the time of hospital discharge. The median gestational age of the 50 (16.5%) preterm infants in the sample was 34.7 weeks (IQR 30.7–36.3, range 23.6–36.9 weeks). The primary seizure etiologies were HIE (43%), ischemic stroke (26%) and ICH (19%).

Table 2 provides the mean results for the six well-being outcome measures total scores and subscales measured at discharge and at 12, 18, and 24 months. HADS anxiety and depression scores were the highest (worst) at discharge compared to the other time points. Frequencies of borderline or abnormal HADS depression scores decreased from 31.5% at neonatal discharge to 11.7% at 24 months, whereas frequencies of borderline or abnormal HADS anxiety scores decreased from 53.2% to 31.5%.

**Figure 1:**
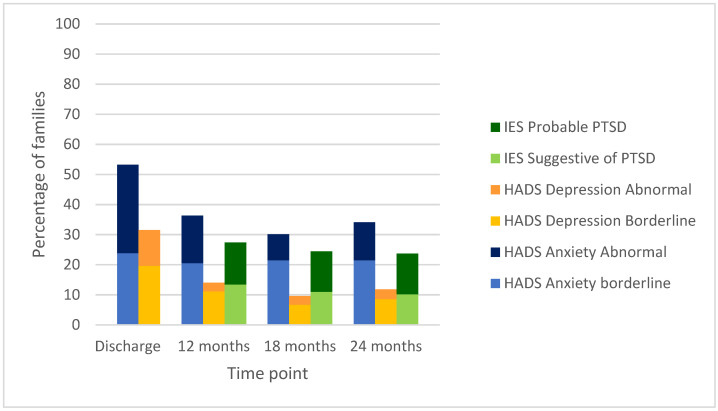
Depression, anxiety and post-traumatic stress symptoms over time for parents of neonates with acute symptomatic seizures.

Frequencies of IES scores above the cut-off suggestive of or probable for post-traumatic stress disorder (PTSD) decreased slightly from 27.4% at 12 months (first measurement) to 23.7% at 24 months (Figure 1). The IOF family impact scores were the highest (worst) at discharge, with little change at the other time points. Neither WHOQOL-BREF quality of life nor the PTGI post-traumatic growth scores differed by time point. Bivariate analysis showed differences in well-being scores based on institution and caregiver respondent (mother, father or legal guardian) but no differences between mother or father respondents (data not shown).

### 3.2. Linear Modeling of Variables Associated with Each Well-Being Outcome

Linear mixed effect regression models for each of the six well-being outcomes, adjusted for institution, time point of measurement and caregiver respondent revealed different associations based on the outcome of interest (Table S1).

#### 3.2.1. Anxiety and Depression

HADS anxiety and depression scores were significantly worse for parents of children with functional impairment compared with parents of children without functional impairment (1.5 points worse for both anxiety and depression; *p* < 0.0001). HADS anxiety and depression scores were nominally worse for parents whose children had been discharged on antiseizure medication at 18 months compared with the 24-month time period (1.0 point worse for anxiety and 0.9 points worse for depression; *p* = 0.031 and *p* = 0.012, respectively). Parents of BIPOC children had nominally better anxiety scores (1.0 points better, *p* = 0.048) compared with parents of white, non-Hispanic children.

#### 3.2.2. Post-Traumatic Stress

Post-traumatic stress scores were significantly worse for parents of children with a diagnosis of epilepsy (7.4 points worse, *p* = 0.0018) compared with parents of children without an epilepsy diagnosis, significantly worse for parents of children receiving developmental support services (3.5 points worse, *p* = 0.0089) compared with parents of children who did not and nominally significantly worse for parents of children with functional impairment compared with parents of children without functional impairment (3.1 points worse, *p* = 0.042). Parents of BIPOC children reported significantly worse post-traumatic stress symptoms compared with parents of white, non-Hispanic children (5.5 points worse, *p* = 0.0033).

#### 3.2.3. Impact on Families

The IOF scores were significantly worse for parents of children with functional impairment compared with parents of children without functional impairment (4.8 points higher, *p* < 0.0001), significantly worse for families with children who had post-neonatal epilepsy compared to families of children without epilepsy (3.8 points worse, *p* = 0.0086) and nominally worse for parents of children who received developmental support services and (1.9 points worse, *p* = 0.015) compared with those whose children did not receive developmental support services. Parents of children who had longer neonatal hospitalizations reported significantly worse impacts on their family (0.5 points worse for each additional week, *p* = 0.0007) compared with parents of children with shorter neonatal hospitalizations.

#### 3.2.4. Quality of Life

WHOQOL-BREF scores were nominally worse for parents of children with functional impairment (5.1 points worse, *p* = 0.011) compared with parents of children without functional impairment. WHOQOL-BREF scores were also nominally worse for parents of children with longer neonatal hospitalizations (0.7 points worse for each additional week, *p* = 0.017) than for parents of children with shorter neonatal hospitalizations.

#### 3.2.5. Post-Traumatic Growth

Parents of BIPOC children had significantly better PTGI scores (9.6 points better, *p* = 0.0049) than parents of white, non-Hispanic children. Parents whose children had public insurance also had nominally better PTGI scores (8.4 points better, *p* = 0.013) than parents of children with private insurance.

### 3.3. Multivariate Modeling of Variables Associated with Parent and Family Well-Being over Time

**Figure 2:**
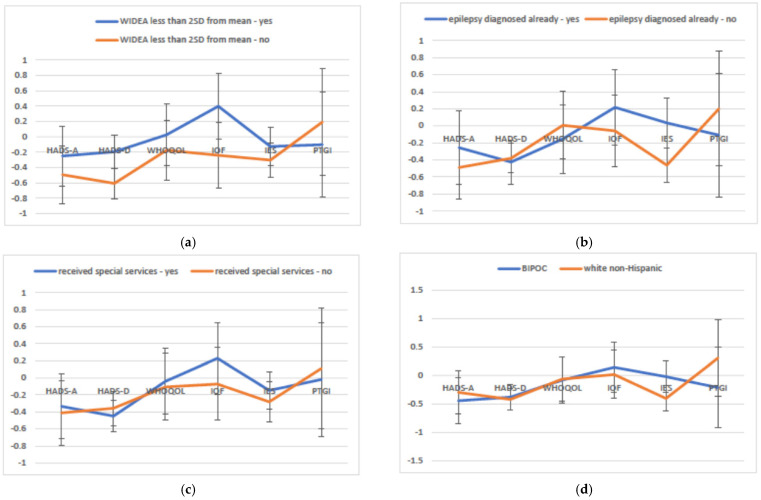
Adjusted mean Z-scores for the variables with significant interactions across the parent mental health and family coping outcomes. Note: The connecting lines are included to make it visually easy to see if the results are the same across outcomes (i.e., the lines are parallel) or if the ordering of the exposure groups changes (i.e., the lines cross). (**a**) Outcomes by child functional impairment (WIDEA-FS > 2 standard deviations below the mean) averaged over all time points (*p* < 0.001); (**b**) Outcomes by child diagnosis of epilepsy averaged over all time points (*p* < 0.0001); (**c**) Outcomes by child receiving developmental support services averaged over all time points (P = 0.002); (**d**) Outcomes by child race/ethnicity averaged over all time points (*p* < 0.0002). HADS = Hospital Anxiety and Depression; IES = Impact of Events; PTSD = Post-Traumatic Stress Disorder; IOF = Impact on Family; WHOQOL-BREF = World Health Organization Quality of Life Brief Assessment; WHOQOL-BREF;PTGI = Post-Traumatic Growth Inventory; Warner Initial Developmental Evaluation of Adaptive and Functional Skills; BIPOC = Black, Indigenous, People of Color.

In the multivariate modeling, we assessed whether the variables of interest had similar or differential effects across all well-being outcomes. After controlling for study site, measurement time point and caregiver respondent, linear mixed modeling revealed significant interactions, all *p* < 0.001 between the following predictors and well-being outcomes, indicating that these variables had differential effects across well-being outcomes: functional impairment, epilepsy diagnosis, child receiving developmental support services, child race/ethnicity and length of stay for child’s neonatal hospitalization. Figure 2 provides the adjusted mean Z-scores for the variables with significant interactions across the well-being outcomes (not shown for length of neonatal hospitalization). Overall, the pattern was similar, with the families of more affected children or of BIPOC children having worse well-being for most outcome measures, except for post-traumatic growth where the pattern was reversed and the groups a with worse well-being outcome reported better post-traumatic growth. The interaction of maternal education and outcomes was nominally significant (*p* < 0.03; see Table S2 for details).

## 4. Discussion

This multicenter study provides a robust characterization of parent mental health and family coping over the first two years after the birth of a child with acute symptomatic seizures. We found that approximately one-third of parents experienced clinically important symptoms of depression at their child’s neonatal hospital discharge and about 10% of parents continued to have depressive symptoms 12 to 24 months later. While more than half of the parents experienced clinically important symptoms of anxiety at their child’s neonatal discharge, almost one-third of parents continued to experience clinically important anxiety 12 to 24 months later. Moreover, clinically important post-traumatic stress symptoms were found in over one-quarter of parents as their child’ reached one-year corrected age, and a similar percentage reported symptoms as their child reached two years corrected age. Impact on the family was moderate and stable over the two-year measurement period, which indicated ongoing stressors and difficulty coping with family life related to the child’s healthcare needs or uncertain prognosis. Notwithstanding, parents reported moderately high quality of life and positive personal change after traumatic stress that was stable over time; however, the wide standard deviations indicate variability in these outcomes. Given the high proportion of parents who experience mental health symptoms at discharge and the risk of persistent parental mental health symptoms, universal mental health education and screening is warranted prior to hospital discharge. Moreover, parent and family coping and resilience should be revisited at children’s and parents’ follow-up primary and specialty care visits to ensure that families are connected to necessary services [7].

We also found significant associations between several clinical factors that differentially influenced parent well-being outcomes over time. Children’s level of functional impairment, diagnosis of epilepsy and need for developmental support services were consistently associated with poorer parental mental health and family coping. Previous research has documented relationships between parent mental health symptoms such as anxiety, depression and post-traumatic stress and poor family functioning when children have neurological morbidities or developmental problems resulting from neonatal conditions [18,19,20], including perinatal stroke [21,22]. Our findings suggest that neonatal seizures be added to this list of risk factors. In contrast to our previous analysis of family well-being at neonatal discharge, we did not find any significant association between specific neonatal seizure etiology, such as HIE, and well-being outcomes. This suggests that the child’s current neurological status and function may have a greater effect on family well-being than the acute cause of their neonatal seizures.

The differential impact of the child’s condition on family well-being over time for children who developed epilepsy is consistent with qualitative research studies that included parents of young children with epilepsy that described the chronic and unpredictable acute nature of the condition and chronic trauma for families [23,24]. Addressing parent mental health concerns early is critically important to the future development, behavior and well-being of children with neurological conditions [21,25,26,27].

Recent systematic reviews of depression [28] and post-traumatic stress [29] in caregivers of children with serious health conditions have found these mental health conditions to be related to a range of family demographic and socio-economic characteristics, caregiver coping styles and factors related to the child’s illness. Our finding of higher PTGI scores, indicating positive personal change after traumatic stress and slightly less depression in parents of BIPOC children, while concurrently reporting worse post-traumatic stress symptoms, warrants further investigation. Previous researchers have reported higher levels of post-traumatic stress in parents of color with ill children due to structural racism and greater overall exposure to stress [30]. Fewer studies have explored factors that protect or buffer against post-traumatic stress; however, these may include social status, practical support and positive coping styles [29].

Our findings should be considered within the context of several study limitations and strengths. First, although we included a broad range of medical and socio-demographic predictors, other unmeasured characteristics such as socioeconomic status or caregiver coping style may also influence family well-being. Although the sample was diverse with respect to child race and ethnicity, we did not have a sufficient sample size to conduct comparisons by specific racial or ethnic groups. Because of the strong influence of structural racism on child and family well-being in the US [31], further studies of the impact of acute neonatal seizures on parent and family well-being over time for specific racial and ethnic groups is needed. Second, children were receiving follow-up care from large tertiary urban medical centers and, therefore, the sample may not be representative of all families of children with neonatal seizures. Finally, we did not measure family access, uptake of mental health or other developmental support services, and these unmeasured interventions may have influenced the findings. Strengths of the study include the large, geographically diverse sample, the longitudinal design and the multivariate analyses that enabled simultaneous consideration of multiple predictors and outcomes and interactions.

## 5. Conclusions

In summary, our findings add to the evidence of the vulnerability of families of children with long-term health conditions and need for greater access to mental health screening and services. Further research regarding interventions to prevent or mitigate threats to family well-being are needed to enable children with neonatal seizures to reach their full developmental potential and quality of life.

## Figures and Tables

**Table 1:** Sample description (n = 303).

	n (%) or Mean (SD)
Male sex	170 (56.1%)
Public insurance	128 (42.3%)
Child race/ethnicity	
Hispanic, any race	47 (15.5%)
Non-Hispanic, Native Hawaiian Pacific Islander	2 (0.7%)
Non-Hispanic, American Indian Alaska Native	2 (0.7%)
Non-Hispanic, Asian	20 (6.6%)
Non-Hispanic, Black/African American	35 (11.6%)
Non-Hispanic, More than 1 race	8 (2.6%)
Non-Hispanic, Other race	6 (2.0%)
Non-Hispanic, White	175 (57.8%)
Unknown	8 (2.6%)
Parent role	
Mother (all time points)	227 (74.9%)
Father (all time points)	27 (8.9%)
Mother and/or father or legal guardian (varied by time)	49 (16.2%)
Maternal education, high school or less	68 (23.5%)
Discharged on medication	194 (64.0%)
Preterm birth	50 (16.5%)
Complex medical condition, includes preterm	79 (26.1%)
Age in days at discharge from hospital, median (IQR)	15 (9–31)
Child diagnosed with epilepsy at 24 months	37 (13.1%)
Child diagnosed with cerebral palsy at 24 months	80 (29.3%)
WIDEA-FS less than 2 SD from mean	
At 12 months	22/187 (11.8%)
At 18 months	49/220 (22.3%)
At 24 months	91/270 (33.7%)
Child receiving developmental support services	
At 12 months	132/187 (70.6%)
At 18 months	140/220 (63.6%)
At 24 months	167/268 (62.3%)

**Table 2:** Well-being over time for 303 parents of neonates with acute symptomatic seizures.

Outcome	Timepoint				
	Discharge n = 143	12 Months n = 171	18 Months n = 209	24 Months n = 246	Change over Time (Controlling for Covariates)
HADS—Depression (0–21; higher scores indicate worse symptoms), mean (SD)	5.6 (4.0)	3.5 (3.2)	3.2 (3.2) ^2^	3.3 (3.1) ^2^	Discharge > 12 m, 18 m, 24 m
					12 m = 18 m = 24 m
HADS—Depression categorical					
Borderline abnormal (8–10)	28 (19.6%)	19 (11.1%)	14 (6.7%)	21 (8.5%)	
Abnormal (>10)	17 (11.9%)	5 (2.9%)	6 (2.9%)	8 (3.2%)	
HADS—Anxiety (0–21; higher scores indicate worse symptoms), mean (SD)	8.3 (4.3)	6.1 (4.3)	5.6 (3.8)	6.0 (4.1)	Discharge > 12 m, 18 m, 24 m
					12 m = 18 m = 24 m
HADS—Anxiety categorical					
Borderline abnormal (8–10)	34 (23.8%)	35 (20.5%)	45 (21.5%)	53 (21.5%)	
Abnormal (>10)	42 (29.4%)	27 (15.8%)	18 (8.6%)	31 (12.6%)	
IES (0–88; higher scores indicate worse symptoms), median (IQR)	n/a	14 (5, 25)	11 (4, 23)	10 (4, 23)	12 m > 18 m
					12 m > 24 m
					18 m = 24 m
IES categorical					
Suggestive of PTSD (24–32)	n/a	23 (13.4%)	23 (11.0%)	25 (10.2%)	
Probable PTSD (>33)		24 (14.0%)	28 (13.4%)	33 (13.5%)	
IOF (15 to 60; higher scores indicate greater impact), mean (SD)					Discharge > 12 m, 18 m, 24 m
Overall	34.8 (9.7)	28.7 (10.7)	28.7 (10.3)	28.0 (10.4)	12 m = 18 m
Financial	10.6 (3.0)	9.6 (3.3)	9.3 (3.4)	9.2 (3.4)	12 m = 24 m
Coping	8.3 (2.5)	8.6 (2.3)	8.9 (2.4)	9.0 (2.8)	18 m >24 m
WHOQOL-BREF (0 to 100 transformed scale; higher scores indicate better QOL) ^1^, mean (SD)					Discharge = 12 m =18 m = 24 m
Overall	73.4 (20.6)	74.8 (18.9)	74.8 (18.8)	76.6 (18.2)	
Physical	70.2 (16.6)	74.5 (16.6)	76.2 (15.3)	75.9 (15.6)	
Psychological	71.6 (18.3)	68.1 (17.8)	69.4 (16.5)	692 (16.7)	
Social	75.4 (19.1)	65.7 (21.6)	66.9 (19.0)	67.2 (19.5)	
Environment	73.1 (16.2)	74.4 (14.8)	74.1 (14.9)	74.0 (14.5)	
PTGI (0–105; higher scores indicate more positive reappraisal), mean (SD)	n/a	63.3 (24.8)	61.1 (27.2)	61.1 (25.5)	12 m = 18 m = 24 m

m = months; HADS = Hospital Anxiety and Depression; IES = Impact of Events; PTSD = Post-Traumatic Stress Disorder; IOF = Impact on Family; WHOQOL-BREF = World Health Organization Quality of Life Brief Assessment; WHOQOL-BREF; PTGI = Post-Traumatic Growth Inventory. ^1^ Transformed scores. ^2^ 18 and 24 m HADS-Depression non-normal distribution; other time points normally distributed.

## Data Availability

Data are presented at clinicaltrials.gov (NCT02789176).

## Supplementary Materials

The following are available online at https://www.mdpi.com/article/10.3390/children9010002/s1, Table S1: Regression coefficients (95% confidence intervals) for linear mixed effect regression models; Table S2: Multi-level modeling of variables associated with parent well-being over time.
