# Supplementary material for: Parent Mental Health and Family Coping over Two Years after the Birth of a Child with Acute Neonatal Seizures
Source: Children (Basel). 2021 Dec 22;9(1):2. doi: 10.3390/children9010002 (PMC8774381; doi:10.3390/children9010002)
Supplement: Supplementary file 1 [file children-09-00002-s001.zip › children-1481846-supplementary.pdf]

Table S1. Regression coefficients (95% confidence intervals) for linear mixed effect regression models, with person-specific intercepts and slope, controlling for institution, time point of measure, and identity of respondent.[1,2]

	HADS-A	HADS-D	IES	IOF	WHOQOL-BREF	PTGI
ASM discontinued (reference = ASM maintained)	0.09 (-1.19, 1.36)	-0.05 (-1.02, 0.92)	--------	--------	--------	--------
CP at 24 months (ref = no CP at 24 months)	--------	--------	--------	--------	--------	--------
Time*ASM maintained<br><br>12 months*ASM discontinued<br><br>18 months*ASM discontinued<br>(ref = ASM maintained, 24 months)	 <br>-0.13 (-1.10, 0.83)<br><br>0.99 (0.09, 1.89)*	 <br>0.18 (-0.56, .092)<br><br>0.89 (0.20, 1.59)*	--------	--------	--------	--------
Time* CP at 24 months<br><br>12 months*CP diagnosis<br>18 months*CP diagnosis	--------	--------	--------	--------	--------	--------
WIDEA-FS < 2SD (ref = WIDEA-FS >2SD below mean)	1.48 (0.76, 2.21))***	1.49 (0.92, 2.05)***	3.08 (0.11, 6.04)*	4.81 (3.04, 6.59)***	5.08 (1.15, 9.00)*	--------
Receiving developmental support services (ref = no services)	--------	--------	3.50 (0.88, 6.12)**	1.93 (0.38, 3.50)*	--------	--------
Diagnosed with epilepsy (ref= not diagnosed)	--------	--------	7.39 (2.77, 12.01)**	3.79 (0.97, 6.60)**	--------	--------
Seizure etiology category	--------	--------	--------	--------	--------	--------
Length of stay	--------	--------	--------	0.47 (0.20, 0.74)***	0.65 (0.12, 1.19)*	--------
Child BIPOC race/ethnicity (ref = White, non-Hispanic)	-1.02 (-2.02, -0.01)*	--------	5.51 (1.86, 9.16)**	--------	--------	-9.64 (-16.32, -2.96)**
Child public insurance (ref = private insurance)	--------	--------	--------	--------	--------	-8.43 (-15.10, -1.77)*
Maternal education	--------	--------	--------	--------	--------	--------

ASM=Antiseizure medication; BIPOC=Black, Indigenous, People of Color; CP=Cerebral Palsy; HADS=Hospital Anxiety and Depression; IES=Impact of Events; PTSD=Post-Traumatic Stress Disorder; IOF=Impact on Family;

WHOQOL-BREF=World Health Organization Quality of Life Brief Assessment; WHOQOL-BREF; PTGI=Post-Traumatic Growth Inventory; ASM=Anti-Seizure Medication; CP=Cerebral Palsy; WIDEA-FS < 2SD=Warner Initial Developmental Evaluation of Adaptive and Functional Skills score more than two standard deviations below the mean for age.

* < 0.05, ** < 0.01, *** < 0.001

[1]Standardized so that higher scores = worse outcomes

[2]All models included time, institution, and who completed the survey (not shown in table)

Table S2. Multi-level modeling of variables associated with parent well-being over time.

**Type 3 Tests of Fixed Effects**				
**Effect**	**Num DF**	**Den DF**	**F-value**	**Pr > F**
Outcome	5	3016	1.67	0.138
Time	2	3016	3.07	0.0467
ASM at discharge	1	3016	0.54	0.4639
CP at 24 months	1	3016	0.6	0.4375
ASM at discharge* Time	2	3016	2.91	0.0545
WIDEA-FS < 2SD	1	3016	18.7	**< 0.0001**
Development support services	1	3016	1.93	0.165
Epilepsy diagnosis	1	3016	1.06	0.3043
Institution	8	3016	0.74	0.6566
Length of neonatal hospitalization	1	3016	4.44	0.0351
White, Non-Hispanic	1	3016	0.05	0.8203
Insurance type	1	3016	0.53	0.465
Maternal education (high school or less)	1	3016	0.12	0.7286
Caregiver role	2	3016	1.73	0.1772
Seizure etiology	3	3016	1.68	0.1693
Outcome*Time[b]	10	3016	2.99	**0.0009**
Outcome*ASM discharge[a]	5	3016	1.5	0.1861
Outcome*WIDEA-FS < 2SD[a]	5	3016	6.42	**< 0.0001**
Outcome* Development support services[a]	5	3016	3.73	**0.0023**
Outcome*Epilepsy diagnosis[a]	5	3016	5.24	**< 0.0001**

Outcome*Institution[b]	40	3016	2.67	**< 0.0001**
Outcome* Length of neonatal hospitalization[a]	5	3016	4.92	**0.0002**
Outcome* White, Non-Hispanic[a]	5	3016	4.89	**0.0002**
Outcome*Insurance type[a]	5	3016	1.54	0.1745
Outcome*Maternal education[a]	5	3016	2.41	**0.0343**
Outcome*Caregiver role[a]	10	3016	11.57	**< 0.0001**

ASM=Anti-Seizure Medication BIPOC=Black, Indigenous, People of Color; CP=Cerebral Palsy; PTGI=Post-Traumatic Growth Inventory; WHOQOL-BREF; Warner Initial Developmental Evaluation of Adaptive and Functional Skills score more than two standard deviations below the mean for age;

[a]Variable of interest

[b]Control variable
